# Number of evaluated lymph nodes and positive lymph nodes, lymph node ratio, and log odds evaluation in early-stage pancreatic ductal adenocarcinoma: numerology or valid indicators of patient outcome?

**DOI:** 10.1186/s12957-016-0983-5

**Published:** 2016-09-29

**Authors:** G. Lahat, N. Lubezky, F. Gerstenhaber, E. Nizri, M. Gysi, M. Rozenek, Y. Goichman, I. Nachmany, R. Nakache, I. Wolf, J. M. Klausner

**Affiliations:** 1 Department of Surgery, Tel Aviv Sourasky Medical Center, 6th Weitzman St., Tel Aviv, Israel; 2 Sackler Faculty of Medicine, The Nicholas and Elizabeth Cathedra of Experimental Surgery, Tel Aviv University, Tel Aviv, Israel; 3 Department of Oncology, Tel Aviv Sourasky Medical Center, Tel Aviv, Israel

## Abstract

**Background:**

We evaluated the prognostic significance and universal validity of the total number of evaluated lymph nodes (ELN), number of positive lymph nodes (PLN), lymph node ratio (LNR), and log odds of positive lymph nodes (LODDS) in a relatively large and homogenous cohort of surgically treated pancreatic ductal adenocarcinoma (PDAC) patients.

**Methods:**

Prospectively accrued data were retrospectively analyzed for 282 PDAC patients who had pancreaticoduodenectomy (PD) at our institution. Long-term survival was analyzed according to the ELN, PLN, LNR, and LODDS.

**Results:**

Of these patients, 168 patients (59.5 %) had LN metastasis (N1). Mean ELN and PLN were 13.5 and 1.6, respectively. LN positivity correlated with a greater number of evaluated lymph nodes; positive lymph nodes were identified in 61.4 % of the patients with ELN ≥ 13 compared with 44.9 % of the patients with ELN < 13 (*p* = 0.014). Median overall survival (OS) and 5-year OS rate were higher in N0 than in N1 patients, 22.4 vs. 18.7 months and 35 vs. 11 %, respectively (*p* = 0.008). Mean LNR was 0.12; 91 patients (54.1 %) had LNR < 0.3. Among the N1 patients, median OS was comparable in those with LNR ≥ 0.3 vs. LNR < 0.3 (16.7 vs. 14.1 months, *p* = 0.950). Neither LODDS nor various ELN and PLN cutoff values provided more discriminative information within the group of N1 patients.

**Conclusions:**

Our data confirms that lymph node positivity strongly reflects PDAC biology and thus patient outcome. While a higher number of evaluated lymph nodes may provide a more accurate nodal staging, it does not have any prognostic value among N1 patients. Similarly, PLN, LNR, and LODDS had limited prognostic relevance.

## Background

Pancreatic ductal adenocarcinoma (PDAC) is the fourth leading cause of cancer-related mortality in the USA [1], exhibiting an aggressive biological behavior with a 5-year overall survival of 7 % [1]. Currently, the only potential curative option for PDAC is surgery followed by adjuvant chemotherapy. Yet, even after this multimodality treatment, disease will recur in over 80 % of these patients [2, 3]. Identification of patients at higher risk of disease recurrence following surgery is of utmost importance and may lead to better allocation of patients to adjuvant therapies.

To date, various prognostic factors have been identified and included in pancreatic cancer staging systems; of them, lymphatic metastasis is constantly considered a powerful indicator of advanced stage and adverse outcome [4–11]. Evaluating lymph node involvement is complex and is affected by the extent of lymphadenectomy as well as the thoroughness of the pathology analysis of the surgical specimen [6, 7]. Currently, lymph node status is defined by the American Joint Committee on Cancer (AJCC) staging system as either negative (N0) or positive (N1) [4–7]. However, several studies suggested that lymph node status can be further refined by either the number of evaluated lymph nodes (ELN) [6, 12–14], number of positive lymph nodes (PLN) [6, 15–18], lymph node ratio (LNR) [6, 13, 19–21], or log odds of positive lymph nodes (LODDS) [19]. Nevertheless, data regarding these nodal staging parameters remain conflicting.

We aimed to evaluate the prognostic value of these nodal staging methods on a relatively large and homogenous cohort of consecutive patients undergoing curative pancreaticoduodenectomy (PD) at a single institution.

## Methods

### Study cohort

The study was conducted at the Tel Aviv Sourasky Medical Center, Tel Aviv, Israel (TLVMC). The Department of Surgery at the TLVMC serves as a national tertiary referral center. Between 1995 and 2013, 317 patients underwent PD with curative intent for PDAC. Seven patients receiving neoadjuvant chemotherapy or radiation were excluded. Data was collected retrospectively (between 1995 and 2010) and prospectively (between 2010 and 2013). Included in the current study are complete clinical and survival data for 282 patients.

The study was approved by the Institutional Review Board (#0052-15-TLV).

### Surgical approach and pathological work-up

We perform a standard lymphadenectomy in patients undergoing PD for suspected PDAC. This includes retrieval of peripancreatic LN and clearance of LN in the hepatoduodenal ligament along the portal vein and hepatic artery to the right side of the celiac trunk and complete clearance of LN on the right side of the upper aspect of the superior mesenteric artery and vein. Our institutional pathologic work-up includes the evaluation of all resected LN. These are completely embedded and labeled according to the International Union Against Cancer TNM LN grouping [22].

### Data collection

The following data was collected for each patient: demographics, tumor characteristics, operative details, pathologic margin status, presence of lymphovascular invasion, perineural invasion, lymph node status, survival data, and oncology treatment. Data on tumor size, ELN, and PLN were obtained from a review of all pathology reports. Staging was determined according to AJCC (sixth edition).

### Lymph node classifications

LNR was determined by dividing the total number of lymph nodes harboring a metastasis by the total number of nodes examined. Patients were sub-classified using previously reported cutoff values of ELN, PLN, or LNR [6–21]. LODDS was calculated as previously described: log [(PLN + 0.5)/(ELN − PLN + 0.5)], 0.5 is added to both the numerator and denominator to avoid singularity [19, 23]. Patients were subdivided into four subgroups according to their LODDS value: LODDS1 (LODDS ≤ −1.5), LODDS2 (−1.5 < LODDS ≤ −1.0), LODDS3 (−1.0 < LODDS ≤ −0.5), LODDS4 (LODDS > −0.5) [24].

### Statistical analyses

The endpoint of this study was overall survival (OS), which was calculated as the elapsed time from pancreaticoduodenectomy to death; data was collected at the time of the last follow-up. Student’s *t* test was used for comparison of continuous variables, chi-square test was used for comparing categorical variables, and Kaplan-Meier curves were constructed to determine OS time. Log-rank test was used to compare OS between subgroups of patients. Univariable Cox proportional hazard regression models were examined to assess the ability of patient characteristics to predict OS. A multivariable Cox model was performed using backward elimination with *p* value cutoff of 0.05. All computations were carried out in SPSS ver. 17 (SPSS Inc., Chicago, IL).

## Results

### Patients and tumor characteristics

During the study period, 282 consecutive patients had PD for PDAC. Patients and tumor characteristics are depicted in Table 1. One hundred and forty-six patients were men (52 %); median age at the time of surgery was 67 years (range, 34–85). Four (1.4 %), nine (3.2 %), and ten (3.5 %) patients died within 30, 60, and 90 days of resection, respectively. Mean tumor size was 3.1 ± 1.6 cm, most patients had T2 or T3 carcinoma (*n* = 225; 80 %), 119 carcinomas (42 %) were categorized as poorly differentiated, and negative microscopic margins were recorded in 221 patients (79 %). Of the 282 patients, 144 patients (51 %) had negative lymph nodes (N0), whereas 138 patients (49 %) had lymph node metastasis (N1). The majority of N1 patients (66 %, *n* = 91) had LNR < 0.3. The mean LODDS value was 0.79 ± 0.47 (range, −1.72 to 1.11). One hundred and eighty patients (63.8 %) received adjuvant chemotherapy, mostly gemcitabine as a single agent (*n* = 129) or in combination with cisplatinum (*n* = 27). Others were treated with 5-FU leucoverin (*n* = 17), FOLFIRI (*n* = 5), and FOLFIRINOX (*n* = 2).

**Table 1:** Patients clinical and pathological characteristics

Variable	No. of patients (%)
Age, years, median (range)	67 (34–85)
Gender	
Male (%)	146 (51.8 %)
Female (%)	136 (49.2 %)
Vascular resection	17 (6 %)
R0 resection	221 (78.4 %)
Average tumor size	3.14 (SD 1.58)
Differentiation	
Well	55 (19.5 %)
Intermediate	86 (30.5 %)
Poor	119 (42.2 %)
Unknown	22 (7.8 %)
Microvascular invasion	
Present	106 (37.6 %)
Absent	123 (43.6 %)
Unknown	53 (18.8 %)
Perineural Invasion	
Present	214 (75.9 %)
Absent	42 (14.9 %)
Unknown	26 (9.2 %)
Lymph node status	
N0	144 (51.1 %)
N1	138 (48.9 %)
ELN, mean	13.5 (SD 5.2)
ELN > 12	82 (29.1 %)
PLN, mean	1.6 (SD 0.9)
LNR ≥ 0.3	47 (34 %)
LODDS, mean	−0.79 (SD 0.47)

### Survival in relation to stage and tumor characteristics

**Fig. 1:**
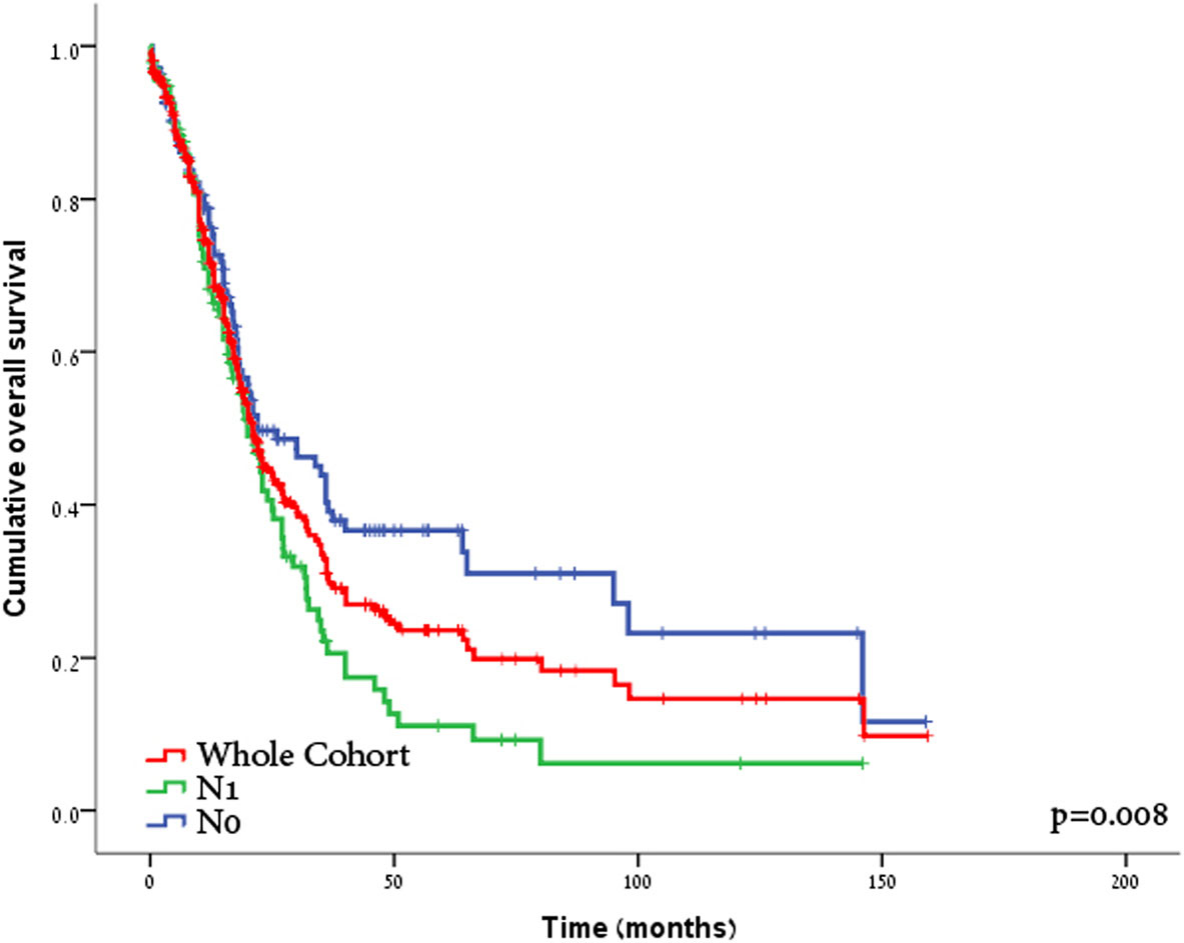
Pancreaticoduodenectomy for ductal adenocarcinoma outcome analysis. Kaplan-Meier overall survival curves for the whole cohort (*red line*) and according to N0 (*blue line*) versus N1 (*green line*) status (*p* = 0.008)

At a median follow-up of 22 months (range, 1–159), the median survival for the entire cohort was 21 months (95 % CI, 17.7–24.2) and the 1-, 3-, and 5-year OS rates are 72, 31, and 23 %, respectively (Fig. 1). Evaluating N1 vs. N0 patients, median survival and 5-year OS rate were 20 vs. 22 months and 9 vs. 34 %, respectively (*p* = 0.008; Fig. 1). Multivariate proportional hazard regression (Cox model) analysis identified tumor size larger than 3 cm (HR 1.29, 95 % CI, 1.05–4.05), poor differentiation (HR 1.58, 95 % CI, 1.14–3.38), lymph node metastasis (N1; HR 3.47, 95 % CI, 1.5–7.36), and positive resection margins (R1; HR 1.41, 95 % CI, 1.03–6.77) as adverse prognosticators (Table 2).

**Table 2:** Predictive factors of survival following PD for PDAC multivariate analysis

Prognostic factor	HR	95 % CI	*p* value
Age > 70 years	1.19	0.77–1.55	0.1
Tumor size > 3 cm	1.29	1.05–4.05	0.01
Tumor differentiation			
Well	1.00		
Moderate	1.23	0.67–3.79	0.26
Poor	1.58	1.14–3.38	0.02
R1 resection	1.41	1.03–6.77	0.05
Lymph node metastasis	3.47	1.50–7.36	0.001
ELN	1.07	0.89–4.75	0.83
PLN	1.39	1.03–6.22	0.12
LNR	0.97	0.71–3.98	0.35
LODDS	1.62	1.11–2.49	0.09
Perineural invasion	1.12	0.63–1.71	0.34
Microvascular invasion	0.97	0.72–1.44	0.48

*HR* hazard ration, *CI* confidence interval

### Survival in relation to the number of evaluated lymph nodes

**Fig. 2:**
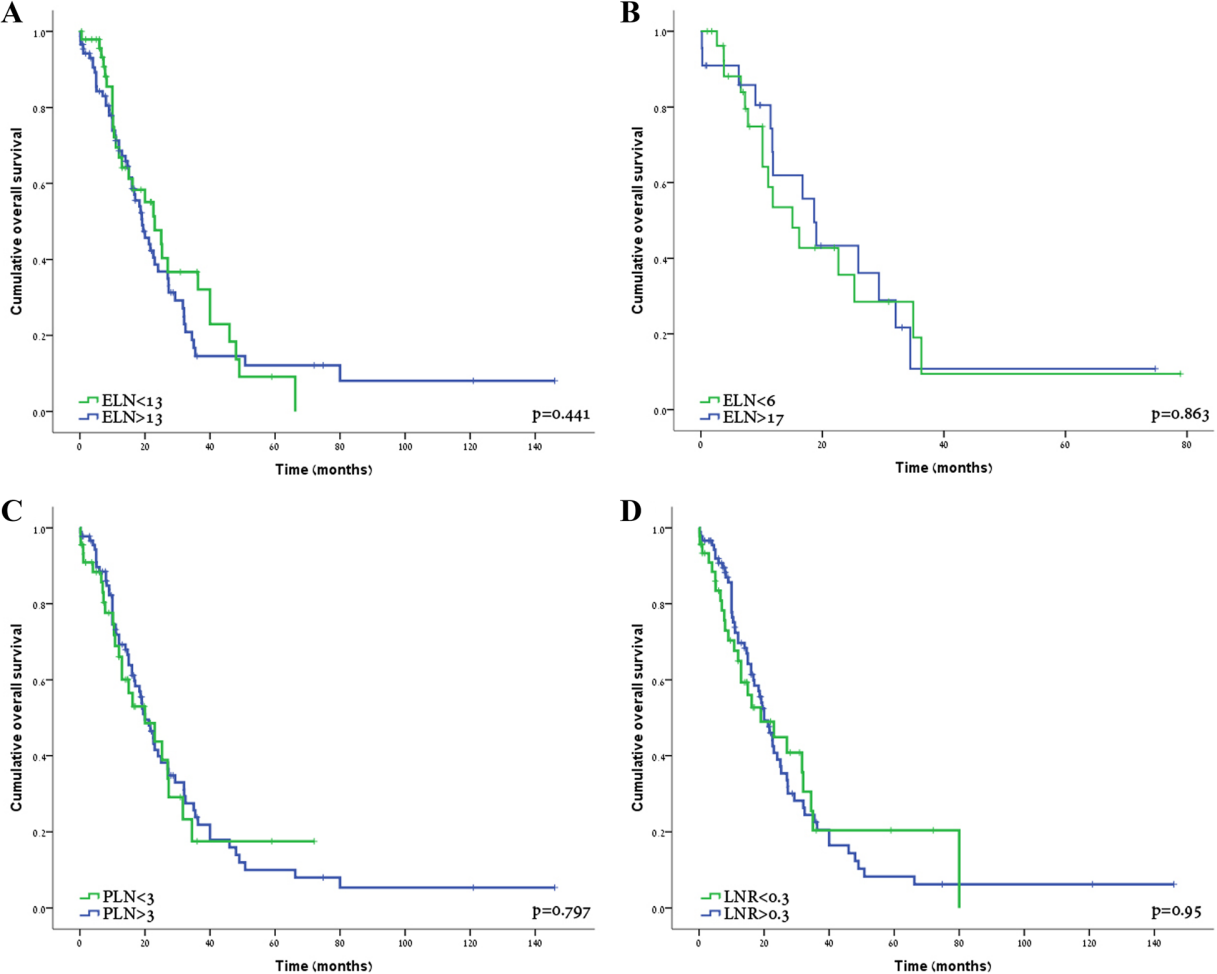
Kaplan-Meier overall survival curves according to ELN < 13 (*green line*) vs. ELN ≥ 13 (*blue line*; **a** *p* = 0.441), ELN < 6 (*green line*) vs. ELN ≥ 17 (*blue line*; **b** *p* = 0.863), PLN < 3 (*green line*) vs. PLN ≥ 3 (*blue line*; **c** *p* = 0.797), and LNR < 0.3 (*green line*) vs. LNR ≥ 0.3 (*blue line*; **d** *p* = 0.95)

The mean number of ELN was 13.5 (range, 1–38) and did not differ over the time course of the study (13; range, 1–35; between 1995 and 2007 vs. 13.8; range, 1–38, between 2007 and 2013; *p* = 0.51). Using a number of cutoff values, Kaplan-Meier survival analyses did not show any association between ELN and survival in N1 patients (Fig. 2a); median survival for the groups of patients with the highest vs. the lowest cutoff values of ELN ≥ 17 (*n* = 28) and ELN < 6 (*n* = 22) were 15 months (95 % CI, 8.1–22) vs. 18 months (95 % CI, 14.3–23), respectively (Fig. 2b, *p* = 0.86).

Although the number of ELN did not predict survival, it correlated with the nodal status. Thus, ELN ≥ 13 was noted in 51 (37 %) of the N1 patients, but only in 31 (22 %) of the N0 patients (*p* = 0.014).

### Survival in relation to the number of positive lymph nodes

Mean PLN was 1.6, with 47 patients (37 %) having three or more PLN. PLN correlated with the number of ELN. Thus, 26 % of patients with >12 ELN had three or more PLN compared to 13 % in the group of ≤12 ELN (*p* = 0.01). Using a cutoff value of 3, Kaplan-Meier survival analysis did not demonstrate a correlation between the number of PLN and survival. As depicted in Fig. 2c, median OS of PLN ≥ 3 and PLN < 3 was similar (20 months in both groups, *p* = 0.8). These results were unrelated to the number of ELN; PLN ≥ 3 did not emerge as a significant prognosticator in either the group of ELN ≥ 17 patients (the highest evaluated ELN cutoff value) or within the ELN < 6 patients (the lowest evaluated ELN cutoff value).

### Survival in relation to the lymph node ratio

The association between LNR ≥ 0.3 and OS was evaluated in the whole cohort of N1 patients. As depicted in Fig. 2d, median survival rates of patients with LNR < 0.3 and LNR ≥ 0.3 were 20 vs. 19 months, respectively (*p* = 0.95). Additional cutoff values were also examined, and none has emerged as a significant predictor of OS.

In order to minimize a potential stage migration effect caused by inadequate LN sampling, we examined the association between LNR ≥ 0.3 and OS in patients with ELN > 12. LNR did not predict OS in these patients, and further analyses failed to identify any minimum number of ELN as a predictor of survival.

### Survival in relation to log odds of positive lymph nodes (LODDS)

**Fig. 3:**
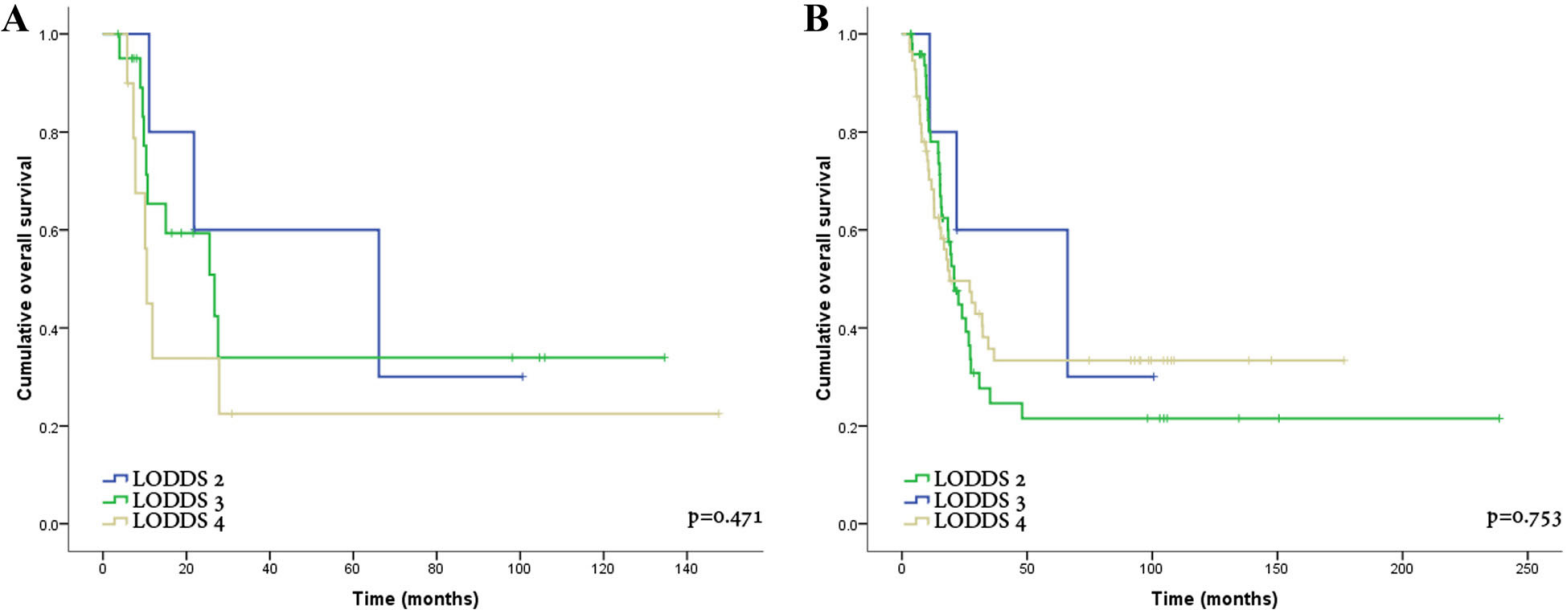
Kaplan-Meier overall survival curves according to LODDS within the whole cohort of N1 patients (**a** *p* = 0.471), LODDS within the group of patients with ELN ≥ 13 (**b** *p* = 0.753)

Patients were categorized into four groups according to their LODDS: LODDS1 (LODDS ≤ −1.5), LODDS2 (−1.5 < LODDS ≤ −1.0), LODDS3 (−1.0 < LODDS ≤ −0.5), and LODDS4 (LODDS > −0.5). All patients categorized as LODDS1 had no LN involvement. Therefore, only the groups LODDS2–4 were included in the analysis. LODDS was not associated with OS in the whole group (Fig. 3a, *p* = 0.47) or in patients with ELN > 12 (Fig. 3b, *p* = 0.75). Additionally, no association between LODDS and OS was noted in patients with LNR ≥ 0.3 or LNR < 0.3.

## Discussion

Numerous studies have shown that involvement of regional lymph nodes is an imperative adverse prognosticator in PDAC [4–11]. Yet, prognosis of patients with N1 disease may vary widely and more accurate classification of these patients may better allocate them to appropriate adjuvant treatment and a better design of clinical trials. Therefore, we examined the association between various nodal classifications and OS in a relatively large cohort of PDAC patients.

A major advantage of the current study is the analysis of a relatively homogenous cohort of patients operated in a single institution by a select number of surgeons experienced in pancreatic surgery. Similarly, the high volume of pancreatic operations performed in our institution (approximately 80 per year) enabled our gastrointestinal (GI) pathologists to gain a vast experience in the evaluation of PDAC pathological specimens of PD. Thus, the combination of adequate surgery and pathological evaluation by trained teams eliminates various possible confounding factors.

Nodal status is affected by the total number of retrieved and evaluated lymph nodes, which is strongly reliant upon adequate lymphadenectomy and a thorough pathological evaluation. Various cutoff numbers have been used to evaluate the prognostic significance of ELN with inconsistent results [6, 12–14]. In agreement with previous studies, our data demonstrate an association between increased ELN and N1 status [6, 7]; yet, ELN by itself did not correlate with OS among N1 patients. The latter observation correlates with the findings of Vuarnesson et al. who demonstrated that an ELN of 16 is required to assess nodal status, yet OS was not affected by the number of evaluated lymph nodes in both N0 and N1 patients [7]. A subset analysis of our N0 patients suggested an association between high ELN and increased OS (data not shown), possibly due to a false classification of N0 patients with a low ELN as node negative. Thus, higher ELN may simply increase the accuracy of nodal staging. These data also suggests that any analysis of N0 and N1 patients grouped together might be biased due to nodal staging inaccuracies and data based on such analyses should be interpreted with caution.

A recent analysis of the SEER data, including over 15,000 patients who were operated for PDAC, suggested an association between ELN, PLN, and LNR with OS [6]. Similarly, several series also suggested an association between ELN and outcomes within N1 patients calling for extended lymphadenectomy in PDAC patients [25–28]. Yet, the role of extended lymphadenectomy in PDAC remains debatable [29–31]. In 1989, Manabe et al. have shown a significant benefit of radical lymphadenectomy arguing that sufficient lymph node clearance is indispensible to cure PDAC patients [27]. More recent data suggest the opposite; in 2005, the results of a prospective randomized trial were published by Farnell et al. who showed no survival difference in standard versus extended lymphadenectomy during PD [29]. Nimura et al. conducted a multicenter RCT in a strict setting, showing that extended lymphadenectomy in radical PD did not benefit long-term survival in patients with resectable pancreatic head cancer [30]. These data indicate that removal of positive lymph nodes from remote basins probably does not affect PDAC patient outcomes. Taken together with our data, it seems that the role of the surgeon in clearing all metastatic lymph nodes is truly limited.

During the last decade, LNR emerged as a prominent nodal prognostic factor in GI cancers, particularly in gastric cancer [6, 13, 19–21, 32, 33]. In accord with this trend, several reports suggested an association between LNR and survival in PDAC [6, 20, 32, 34–38]. Not surprisingly, the cutoff LNR values suggested by these papers are inconsistent: Showalter el al. have shown that LNR > 33 % is associated with adverse prognosis [16], Ashfaq et al. demonstrated that PDAC patients with LNR > 0.1 have shorter OS [12], whereas Liu et al. suggested the cutoff of LNR ≥ 0.4 as a predictor for worse outcome [13]. Many of these studies included N0 patients in their analysis, for whom LNR is irrelevant. Our data suggest no association between LNR and survival. This may be attributed to the exclusion of N0 patients from the current analysis. In agreement with our findings, Kang et al. analyzed a cohort of nearly 400 PDAC patients and also failed to show an association between PLN and LNR and prognosis [15].

LODDS is a novel indicator of lymph node status, developed with the aim of improving the accuracy of nodal classification for prognostic assessment. LODDS has several theoretical advantages over LNR. Mathematically, this logarithmic function distinguishes between patients with LNR = 0 (N0 patients) as well as patients with similar LNR but different PLN and/or ELN values (i.e., 2/4 and 5/10). LODDS has been investigated as a prognostic factor in several cancers [38–42]; however, data concerning its predictive role in pancreatic cancer is scarce. La Torre et al. analyzed a series of 143 PDAC patients who had pancreatic resection. They concluded that LODDS and LNR are more powerful predictors of survival than lymph node status and that LODDS allows better prognostic stratification compared with LNR in node-negative patients [19]. The results of the present study question the potential role of LODDS as a prognostic factor in N1 PDAC patients, unrelated to nodal status, ELN, or LNR.

The major limitation of the present study is its retrospective nature. All nodal parameters were dependent on past evaluation of dedicated pathologists that worked with our surgical team using different techniques over a period of almost two decades. In addition, surgical adequacy, as well as patient selection for PD, has changed over this time period. Another potential weakness is that the average number of evaluated lymph nodes is relatively low compared to several other studies [6, 36, 43]; yet, our subset analyses in patients with ELN > 12 and even in a subgroup of patients with ELN > 15 did not alter our results.

Since not all patients included in the present study cohort have been treated with post-operative chemotherapy, the results might be biased. Adjuvant chemotherapy has become the standard of care for PDAC patients only after the publication of the CONKO-001 trial in 2007. Per institutional guidelines, no patient received adjuvant therapy prior to that period, while since 2008, all patients received treatment, regardless of tumor stage. While some deviations from guidelines may occur, it is still likely that administration of adjuvant treatment is not a major confounding factor in this study.

Lastly, data concerning post-recurrence therapy was not collected and is not included in our analysis. While the pattern of oncology therapy for PDAC has changed dramatically during the study period (gemcitabine in 1997, FOLFIRINOX since 2011), the median overall survival of patients with metastatic disease increased from about 5 months to only 11 months. Therefore, any potential effects of the first- and second-line therapy on survival are small. Moreover, all patients were treated at our institution according to similar guidelines and treatment for metastatic disease was not affected by nodal status at presentation. Therefore, it is likely that the subsequent oncology treatments did not significantly affect our conclusions regarding the role of lymph node involvement.

## Conclusions

Pancreaticoduodenectomy with conventional lymphadenectomy remains the primary method of locoregional control of PDAC and is the only potentially curative treatment for this disease. Our present study demonstrates that the presence of lymph node metastasis is an independent adverse prognosticator, regardless of the total number of ELN, number of PLN, LN ratio, or LODDS of positive lymph nodes. The data does not refute the role of adequate lymphadenectomy or the need for a meticulous evaluation of lymph node status by the pathologist. However, it implies that any presence of nodal involvement represents a very aggressive biological behavior of the primary tumor. Clearly, further clinical and molecular investigation is needed in order to augment our present understanding of lymphatic metastasis biology and to better understand its impact on PDAC patient outcome.
